# Recovery strategies following COVID-19 disruption to cervical cancer screening and their impact on excess diagnoses

**DOI:** 10.1038/s41416-021-01275-3

**Published:** 2021-02-09

**Authors:** Alejandra Castanon, Matejka Rebolj, Francesca Pesola, Peter Sasieni

**Affiliations:** King’s College London, Faculty of Life Sciences & Medicine, School of Cancer & Pharmaceutical Sciences, London, UK

**Keywords:** Epidemiology, Cancer screening, Outcomes research

## Abstract

**Background:**

The COVID-19 pandemic has disrupted cervical cancer screening services. Assuming increases to screening capacity are unrealistic, we propose two recovery strategies: one extends the screening interval by 6 months for all and the other extends the interval by 36/60 months, but only for women who have already missed being screened.

**Methods:**

Using routine statistics from England we estimate the number of women affected by delays to screening. We used published research to estimate the proportion of screening age women with high-grade cervical intraepithelial neoplasia and progression rates to cancer. Under two recovery scenarios, we estimate the impact of COVID-19 on cervical cancer over one screening cycle (3 years at ages 25–49 and 5 years at ages 50–64 years). The duration of disruption in both scenarios is 6 months. In the first scenario, 10.7 million women have their screening interval extended by 6 months. In the second, 1.5 million women (those due to be screened during the disruption) miss one screening cycle, but most women have no delay.

**Results:**

Both scenarios result in similar numbers of excess cervical cancers: 630 vs. 632 (both 4.3 per 100,000 women in the population). However, the scenario in which some women miss one screening cycle creates inequalities—they would have much higher rates of excess cancer: 41.5 per 100,000 delayed for screened women compared to those with a 6-month delay (5.9 per 100,000).

**Conclusion:**

To ensure equity for those affected by COVID-19 related screening delays additional screening capacity will need to be paired with prioritising the screening of overdue women.

## Background

Cervical screening aims to identify abnormal cells in the cervix and treat them before they progress to cancer. In England, cervical screening is mostly carried out in general practice by specially trained nurses. Until December 2019, when the national roll-out of human papillomavirus (HPV) primary testing was completed, screening was done using liquid-based cytology. Screening has been offered to women aged 25–49 years at 3-yearly intervals (although the plan is to extend to 5-yearly with HPV testing) and to those aged 50–64 years at 5-yearly intervals. The age-appropriate coverage is ~72% of the eligible women.^1^ In recent years, the European age-standardised incidence of cervical cancer in England for women aged 25–64 years has been hovering ~9.5/100,000.^1^

Vaccination against HPV (bivalent vaccine) was introduced in England in 2008–2009 to girls aged 12–13 years (born from 1st of September 1995 to 31st of August 1996) and to a catch-up cohort aged 14–18 years (born from 1st of September 1990 to 31st of August 1995). Coverage among girls aged 12–13 years has remained ~86% since.^2^

Due to the COVID-19 pandemic, cervical cancer screening has been severely disrupted the world over. In England, invitations for screening were suspended from April 2020.^3^ Although call/recall was reinitiated in June, primary care providers were given the option to delay invitations for screening for up to 6 months if necessary.^4^ Similar disruptions to screening have been reported in other countries.^5,6^ It is likely that the second and future waves of the pandemic will result in further disruption to screening.

As the pandemic is not yet over, it is currently unknown how quickly primary care and laboratories will be able to restore cervical screening services to pre-pandemic levels, what capacity will be available to address the screening backlog that has accumulated since GP practices closed for non-urgent face to face contact in March 2020 and how many women will be willing to take up their screening invitation in the post-COVID-19 era. Nevertheless, it is possible to estimate the size of the effect that a disruption like this is likely to have on cancer incidence, depending on the screening programme’s approach to compensate for the lost opportunity to be screened.

Assuming that the health service will not be able to increase screening capacity considerably compared with previous years, it will be difficult or impossible to “catch-up” with the backlog of screening. Rather, the service must choose between extending the screening interval for a whole round of the programme or trying to confine the disruption to women who have already missed being screened. Here, we model the impact of delays to cervical cancer screening on the excess diagnosis of cervical cancers among women of screening age (25–64 years) in England.

## Methods

The effect of a delay in attendance to cervical screening is explored under two scenarios. For both scenarios, the impact of COVID-19 on the provision of cervical cancer screening would affect services over one screening cycle (3 years in women age 25–49 years and 5 years in women aged 50–64 years) only. The length of the COVID-19-related disruption is the same in both scenarios (6 months), but the delay to screening thereafter and hence the proportion of women affected by the delay differs in each recovery scenario (i.e. 6 months in the first scenario and 36/60 months, depending on age, in the second scenario). Both scenarios assume that follow-up of women testing positive at screening before the disruption was followed up on time and that follow-up services do not experience delays once the disruption ends.

The first scenario considers a rolling delay of 6 months for all women in England over one screening cycle. This means that for a single screening cycle, women would be invited for HPV testing at 3.5 or 5.5 years (i.e. 6 months delay regardless of age) after their previous invitation to cytology screening, depending on their age. They would resume with a standard interval thereafter. The age at which screening is offered would be permanently increased by 0.5 years (up to age 65.5 years). Young women entering the programme after the disruption has been resolved would not be affected.

The second scenario assumes invitations to screening were likewise disrupted for a 6-month period and that during the recovery period women who were due a screen during the COVID-19-related disruption do not receive screening during this cycle (i.e. a 36- or 60-month delay depending on age). Women whose invitation was not affected by COVID-19 disruption would continue to be invited as normal.

Both scenarios assume no disruption to screening uptake once screening resumes.

### Population estimates

The age-specific numbers of women screened in England following an invitation for screening (in categories: “call” (first ever invitation to screening), “recall” (second and later routine invitations to screening) and “outside the programme” (predominantly women who were invited but attended 12 months or more after the invitation is issued)) as reported in the NHS Cervical Screening Programme statistics for years 2018–2019^7^ were used to estimate the number of women by age group who would have normally been routinely screened over a 12-month period. In the first scenario, the number of women screened in 1 year was multiplied by 3 or 5 depending on the age group to obtain the total population affected by the delay (*n* = 10,699,491). In the second scenario, the affected population was half of those attending over 12 months (*n* = 1,522,219).

The national statistics was the source of data for the estimated size of the female population in mid-2019 in England by age group.^8^

### Population with cervical intraepithelial neoplasia

To estimate the number of screened women with a high-grade cervical intraepithelial neoplasia (CIN) detectable through HPV screening, data reported from the first round of screening in the English HPV primary screening pilot was used, where by far the majority of women were unvaccinated.^9^ The pilot reported that 6.6% women at the age of 25–29 years, 1.6% at the age of 30–49 years and 0.5% at the age of 50–64 years had a CIN grade 2 or worse detected following HPV primary screening, either at baseline or at one of two early recalls.

The proportion of women estimated to have high-grade CIN was multiplied by the total population affected by the disruption, in order to obtain the number of women in whom CIN2 or worse diagnoses were delayed because screening could not take place as scheduled. Estimates of the population affected were adjusted for the proportion of women aged 25–29 and 30–34 years who were vaccinated with three doses. The proportions of the birth cohort who were vaccinated (and the age of vaccination) were taken from the national statistics^10^ and are presented in Supplementary Table 1. The odds ratios (ORs) of being diagnosed with a CIN grade 3 or worse by age at vaccination as reported for Scotland^11^ (comparing to cohorts that were not offered vaccination) were used to adjust the proportion of high-grade CIN per 100,000 screened women to better reflect the detection in vaccinated cohorts. Compared to unvaccinated women, the odds of high-grade CIN were the lowest (OR = 0.14) among those who were vaccinated at ages 12 or 13 years. The OR increased thereafter to 0.18 in those vaccinated age 14 years, 0.29 at age 15 years, 0.27 at age 16 years, 0.55 at age 17 years and 0.85 at age 18 years.

### Progression of CIN

The proportion of high-grade CIN that would have progressed to cervical cancer in 6 months was estimated using progression estimates from the Landy et al.^12^ modelling study. In that study, parameter sets were chosen to be consistent with the literature. They reported 6-monthly progression rates from high-grade CIN to asymptomatic cancer of 0.12% for women aged <30 years, 0.25% at age 30–34 years, 0.35% at age 35–39 years, 0.65% at age 40–49 years, 0.9% at age 50–61 years and 1.1% at age 62 years or more. For the second scenario, cumulative transition probabilities (following an exponential distribution)^13^ up to 36 months (for women aged 25–49 years) or 60 months (for those aged 50–64 years) were calculated from the same 6-monthly progression rates to estimate the cumulative number of women whose undetected CIN would have progressed to cervical cancer during one age-appropriate screening round (Supplementary Table 2).

## Results

Delaying screening for 6 months for the whole population (scenario 1) would result in ~10.7 million women being affected by the disruption, whereas delaying screening for an entire screening cycle (scenario 2) only to those directly affected would impact 1.5 million women (Table 1). However, both scenarios would result in very similar expected numbers of excess cancers, ~630 over one screening round. These cases amount to ~4 additional cases per 100,000 women in the general population over one screening cycle.

**Table 1:** Excess cancers due to delays in screening. Scenario comparison.

	Scenario 1^a^	Scenario 2^b^
Total population in England	14,677,008	14,677,008
Population affected by delayed screening	10,699,491^c^	1,522,219^d^
Excess cervical cancers	630	632
Rate of excess cervical cancer per 100,000 screened women	5.9	41.5
Rate of excess cervical cancer per 100,000 population	4.3	4.3

^a^Scenario 1 considers a rolling delay of 6 months for all women in England over one screening cycle.

^b^Scenario 2 assumes women who experience COVID-19-related disruptions to screening have a 36- or 60-month (depending on age) delay to their screening.

^c^Women who undergo screening in one screening round of 3 or 5 years, depending on age, estimated from the NHS Cervical Screening Programme statistics for years 2018–2019.

^d^Women who undergo screening during a period of 6 months, estimated from the NHS Cervical Screening Programme statistics for years 2018–2019.

When only taking into account the women who were affected by the delay (i.e. those that would normally have participated in screening but were unable to do so, or could only do it with a delay), the excess cervical cancer incidence rates differed vastly between the two scenarios. Those who miss an entire round (scenario 2) would have seven times higher rates of excess cancer compared with those whose screening was delayed by 6 months (41.5 per 100,000 women compared to 5.9, respectively, Table 1).

The age-specific distribution of excess cancer diagnoses with a 6-month delay can be found in Table 2, and the results for 36- or 60-month delay are shown in Table 3. In both scenarios, women aged 40–49 years are expected to be the most affected, whereas the impact on women aged 25–34 years is mitigated by vaccination against HPV 16 and 18.

**Table 2:** Scenario 1. Population affected and excess cancers given a rolling 6-month delay to screening over one screening cycle.

Age group in 2020	Women affected by a 6-month rolling delay to screening^a^	Estimated number of women with CIN2/3 in population	Excess diagnoses of cervical cancer	Excess rates per 100,000 affected	Excess rates per 100,000 population
25–29	1,533,957	45,772^b^	55	3.6	2.9
30–34	1,329,927	18,952^b^	47	3.6	2.5
35–39	1,365,366	21,846	76	5.6	4.1
40–44	1,238,154	19,810	129	10.4	7.5
45–49	1,316,637	21,066	137	10.4	7.3
50–54	1,698,585	8493	76	4.5	3.9
55–59	1,284,705	6424	58	4.5	3.1
60–64	932,160	4661	51	5.5	3.2
Total	10,699,491	147,024	630	5.9	4.3

^a^ Women who undergo screening in one screening round of 3 or 5 years, depending on age, estimated from the NHS Cervical Screening Programme statistics for years 2018–2019.

^b^Rate of CIN2/3 in the population adjusted for proportion vaccinated.

**Table 3:** Scenario 2. Population affected and excess cancer due to women having their screen delayed by 3 or 5 years, depending on age.

Age group in 2020	Women affected by delay to screening^a^	Estimated number of women with CIN2/3 in population	Excess diagnosis of cervical cancer	Rates per 100,000 affected (all stages)	Rates per 100,000 population (all stages)
25–29	255,660	9427^b^	68	26.5	3.6
30–34	221,655	3407^b^	51	22.9	2.7
35–39	227,561	3641	76	33.2	4.0
40–44	206,359	3302	126	61.2	7.4
45–49	219,440	3511	134	61.2	7.2
50–54	169,859	849	73	43.0	3.7
55–59	128,471	642	55	43.0	3.0
60–64	93,216	466	49	52.1	3.1
Total	1,522,219	25,246	632	41.5	4.3

^a^Women who undergo screening during a period of 6 months, estimated from the NHS Cervical Screening Programme statistics for years 2018–2019.

^b^Rate of CIN2/3 in the population adjusted for proportion vaccinated.

## Discussion

Cervical screening at regular intervals prevents cervical cancer.^14–16^ The length of the interval typically represents the period during which the benefit of screening is observed.^15^ Hence, any delays to cervical screening will negatively impact cancer diagnoses. Both scenarios modelled here resulted in ~630 excess cancers, equivalent to just over 4 per 100,000 women in the population, distributed across one round of screening (i.e. 3 or 5 years depending on age). However, the risk of cervical cancer to an average woman who would have attended screening is seven times higher if they had to delay their screening for a whole screening round than if they had to delay screening for only 6 months.

It will be challenging to assess the impact of COVID-19 related delays by monitoring population rates of cervical cancer. The impact at a population level will be spread over a number of years. Further, given the roll-out of HPV primary screening in 2020 an initial increase in cancer diagnoses is expected (because the test is more sensitive than cytology).^17^ Evaluation of screening histories from women diagnosed with cervical cancer once screening services resume will provide the best evidence of the actual impact of COVID-19 delays to cervical screening.

Our model can be applied to a variety of situations that have the ability to derail a cervical screening programme in the same way as has happened with the COVID-19 pandemic. A limitation of our analysis is that we had to rely on the indirectly estimated parameter for the rate of progression of CIN to cervical cancer. This is not unusual in modelling studies, but the parameters used in this study had been previously calibrated to replicate cancer rates in England.^12^ We have not taken into account the effect of any delays in diagnostics and treatment of women with a positive screen, nor any drop in the coverage once screening fully resumes; both would result in additional cases of cervical cancer. Finally, we focused on the additional burden of cancer due to the disruption. Any worsening of the prognosis of screen-detectable cancers due to a delayed diagnosis was beyond the scope of our analysis, but would further worsen the impact of the COVID-19 disruption.

Our results show that the overall population burden of cervical cancer does not depend on whether scenario 1 or scenario 2 takes place. The two scenarios, however, clearly differ in terms of how this burden is distributed among the population. Scenario 2 culminates in a substantially higher excess risk per affected woman. Because the CIN2/3 lesions left undetected would have a longer time to progress (compared to scenario 1), it is also more likely that the excess cancers under this scenario would be diagnosed at later stages. In the name of equity, therefore, our analysis calls for measures that ensure that women do not miss an entire screening round on account of the COVID-19 disruption, that is, scenario 2 should be avoided and scenario 1 would be preferable. Under naive assumptions used in our model, this means that an additional nine million women would be affected by the shorter disruption that is also not without a risk. However, this risk could be diminished in the programmes by (a temporary) increase in the screening capacity in order to clear the screening backlog (on top of the usual workload). If, for example, the screening capacity increased by 33%, it would still take 18 months to clear the 6-month backlog, but it would significantly decrease the total number of excess cancers. We note that a small increase in capacity is assumed in scenario 1 to ensure that women entering screening for the first time are not affected. Unfortunately, the demand for reagents to carryout HPV testing competes directly with the demands for COVID reagents and hence increasing screening capacity may not be feasible.

Increasing the screening capacity alone will not be sufficient. Making sure that women feel confident enough to attend for screening when they are due should be another priority. This is, again, highlighted by the seven-fold higher excess risk of cervical cancer among those who are unable or unwilling to access screening for a whole round, and highlights the importance of messaging to encourage women overdue their screen to attend as soon as possible. Unfortunately, it is often difficult for primary care providers to assess a woman’s prior screening history at the time when they are offered a screening appointment. Nevertheless, consideration should still be given to strategies that will allow the identification and prioritisation of screening of women affected by the COVID disruption to ensure that cervical screening remains a truly equitable service.^18^

## Supplementary information

Supplemental Tables

## Data Availability

Source data for this study is freely available in published literature.
